# An Abnormal Case of Pneumonia

**Published:** 1898-03-19

**Authors:** 


### AN ABNORMAL CASE OF PNEUMONIA.

In a clinical lecture by Sir William Broadbent, reported in the *British Medical Journal*, a good account is given of a case of pneumonia which ran an abnormal course and placed the patient in great peril of his life. The fact is insisted on that normal pneumonia tends to terminate by crisis. The temperature falls, the pulse quiets down, the respiration becomes less frequent, the tongue cleans, and a tranquil sleep brings to an end the delirium of stupor, the muscular tremor, or twitching, or other evidence of nervous prostration or disturbance which may have supervened. But the crisis relates to symptoms only, not at all to physical signs, and, while the patient may have all the appearance of convalescence, dulness, tubular breathing and bronchophony are found to be as distinct as ever, and a week or ten days may be occupied in removing the exudation from the air cells. Clearly, this crisis is the manner in which the normal and uncomplicated disease comes to an end, and each day of delay in its appearance must bring increased anxiety as to the result. When it is postponed beyond the ninth day it may usually be concluded that something underlies the pneumonia such as tubercle or septicæmia; but even more serious in its import than a delay in the crisis of pneumonia is an abortive attempt at crisis. "Nature has made her effort and failed," and there is nothing more to be expected from the constitution.

This then was the state of affairs in the case related. Instead of the crisis, with rapid fall of temperature to normal or subnormal, and a general amelioration in the symptoms, the temperature only fell to 103 deg. F., and the boy, instead of being better, was obviously worse in all respects. He became delirious, the pulse went up to 144 deg., and the respirations to between 50 and 60 deg.; there was a deep circumscribed flush on each cheek, and the temperature rose again. On examination the extraordinary fact was noticed that, instead of dulness, there was resonance everywhere, air penetrated freely every part of the lung, and neither rhonchus, nor sibilus, nor redux crepitation could be detected anywhere. At no point was there a trace of the consolidation which less than 48 hours before had involved nearly the whole of the lung.

Nothing could be more grave than the condition of affairs thus revealed. Two days previously the air vesicles of a great part of the lung had been choked with exudation, which had in that short space of time entirely disappeared. It had not been expectorated or swallowed; it could only have been absorbed; and there was every reason to fear that it was playing the part of a poison in the blood, for it was evident that the patient was under some toxic influence.

An interesting observation made was that, while the degree of fever was sufficient to explain the frequency of the pulse, there was nothing in the state of the lung or the heart to explain the inordinate frequency of the breathing, viz., 60 to 64 respirations in the minute. This could only be due to the effect of some poison acting on the nervous system, or, as was suggested by the sequel, to some pressure at the root of the lung.

As the temperature was still rising the patient was freely sponged with cold water, and an ice bag was applied to the head. This, however, had but little effect, and the temperature continued to rise, in a short time reaching 105·6 deg. F. Upon this the patient was enveloped in a dripping sheet, the moisture of which was continually renewed by droppings of ice-cold water from a sponge. After about 25 minutes some impression began to be made on the temperature, the flush on the cheeks paled, and when this became very decided the wet sheet was withdrawn, the patient, still naked, being covered by a single blanket. The temperature fell to about 103 deg., but after a few hours the wet sheet had to be applied again. Five grains of quinine were given after the first wet pack, and again later in the day. "There can be no doubt that the wet sheet saved the boy's life. He was getting visibly worse in every respect from hour to hour, and was apparently drifting rapidly into hyperpyrexia."

Now for the explanation. From this time the temperature fell, always, however, touching 101 deg. at some period of the 24 hours. The pulse remained frequent, and the respirations maintained a great relative rapidity. Cough persisted, although no physical signs to account for it could be found. After about a week the temperature began again to rise, and the paroxysms of cough became quite uncontrollable, lasting for two or three hours at a time. The cough was like that of whooping-cough, and from time to time provoked vomiting. Talking, eating, and turning on the side provoked paroxysms. There could be no doubt about the meaning of a cough of this character; it could only be due to enlarged bronchial glands. Irritation and inflammation of the glands at the root of the lung had obviously been set up by the material absorbed so rapidly from the lung. At last a slight rigor and another rise of temperature announced suppuration, and shortly after this it was found that the apex of the heart was pushed over to the left. Evidence of pressure upon the root of the lung was also furnished by imperfect entry of air into the right lung, with distant tubular breathing in the first space near the sternum, and slight impairment of resonance in the right interscapular space about the level of the spine of the scapula. At last, after a very anxious time, pus was expectorated, but at first in only a very small quantity. The right base became dull and it seemed as if the mediastinal abscess were about to burst into the pleura; there were also profuse night sweats and suppurative fever. Then a second expectoration of pus, and finally a third, after which no more was seen. After that all went on well, and all that remained appeared to be a scarcely recognisable impairment of resonance and respiratory murmur at the right base.

The first point of interest in this case is that the boy recovered from so serious a condition; the next seems to us to lie in the exceeding efficacy of the cold sheet, and in the fact that it evidently did something more than merely reduce temperature; and, lastly, we have in this case a most useful and important illustration of one of the ways in which abnormal pneumonia may kill. If this boy had died at the crisis, during the period when the pneumonic effusion was being absorbed, and its products poured as a poison into the blood by way of the lymph stream, there would probably have been nothing more than swollen and "irritated" glands to show what was going on. But the after history showed pretty plainly that absorption was taking place through the lymphates, and that a condition of true "blood-poisoning" was taking place.

